# Assessing healthcare service quality using routinely collected data: Linking information systems in emergency care

**DOI:** 10.1007/s10916-020-01572-z

**Published:** 2020-05-08

**Authors:** Harald Dormann, Patrick Andreas Eder, Henner Gimpel, Oliver Meindl, Asarnusch Rashid, Christian Regal

**Affiliations:** 1 Department of Emergency Medicine, Klinikum Fürth, Fürth, Germany; 2 Zentrum für Telemedizin Bad Kissingen, Bad Kissingen, Germany; 3 Project Group Business & Information Systems Engineering of Fraunhofer FIT, Augsburg, Germany; 4 FIM Research Center, University of Augsburg, Augsburg, Germany

**Keywords:** Healthcare service quality, Quality measurement, Quality indicator, Emergency department, Routinely collected data

## Abstract

Emergency departments need to continuously calculate quality indicators in order to perform structural improvements, improvements in the daily routine, and ad-hoc improvements in everyday life. However, many different actors across multiple disciplines collaborate to provide emergency care. Hence, patient-related data is stored in several information systems, which in turn makes the calculation of quality indicators more difficult. To address this issue, we aim to link and use routinely collected data of the different actors within the emergency care continuum. In order to assess the feasibility of linking and using routinely collected data for quality indicators and whether this approach adds value to the assessment of emergency care quality, we conducted a single case study in a German academic teaching hospital. We analyzed the available data of the existing information systems in the emergency continuum and linked and pre-processed the data. Based on this, we then calculated four quality indicators (Left Without Been Seen, Unplanned Reattendance, Diagnostic Efficiency, and Overload Closure). Lessons learned from the calculation and results of the discussions with staff members that had multiple years of work experience in the emergency department provide a better understanding of the quality of the emergency department, the related challenges during the calculation, and the added value of linking routinely collected data.

## Introduction

Emergency departments (EDs) have the liability to deliver high-quality emergency care. This requires continuously performing structural improvements, improvements in the daily routine, and ad-hoc improvements in everyday life.

However, EDs are barely capable of fully implementing this into the daily business. One reason is that many different actors across multiple disciplines within the emergency care continuum (ECC) collaborate to provide emergency care (e.g., paramedics, hospital staff). As a consequence, patient-related data is stored in several information systems (IS). Due to this distributed storing of data, many EDs struggle to establish quality indicators (QI) that holistically assess the quality of emergency care. Most of the current QIs focus either on particular aspects of the ED (e.g., specific diagnosis or patient groups), have to be calculated manually, or require a collection of additional data [1]. To address this issue, we aim to link and use routinely collected data of the different actors within the ECC. This leads to a reduction of efforts and costs for the data preparation and QI calculation. However, the usage of this data is challenging due to various issues (e.g., data heterogeneity, lack of structured data, and fragmentation across various information systems).

Our approach relates to other German and international initiatives on using routinely collected data to increase emergency care quality. An example of a German initiative is the TRUST project, which provides trend and structural analyses of the rescue service in Bavaria using routinely collected data (i.e., structural, operational, and accounting data of the rescue service) [2]. Another German project is AKTIN that aims to develop an interoperable emergency registry by providing a distributed infrastructure that makes routinely collected data usable for health services research to foster quality management within EDs [3]. However, both initiatives mainly focus on a macro perspective (e.g., general care situation in a region) and do not aim to identify the quality, and thus improvement potentials, within a specific hospital or ED. Other European initiatives focus on the development of new QIs [4–7] or calculate specific QIs based on additionally collected data (e.g., from local registers) [8–11]. Besides, there exist initiatives that focus on the use of routinely collected data like the MIPS system in the United States, for example, which requires clinicians to calculate 268 QIs [12]. However, only 14 of these QIs are associated with the ED and primarily refer to specific diagnoses or patient groups. Therefore, they provide limited value to identify improvement areas regarding the structure or routine of the ED.

Overall, first attempts exist that link data within the ECC to improve emergency care. However, to the best of our knowledge, no research uses routinely collected data within the ECC to assess QIs of an ED in order to derive general improvement potentials (i.e., independent of specific diagnoses or patient groups) or ad-hoc improvements in everyday life. Hence, in this research, we conduct a case study in a German ED and aim to answer the following research questions:

- *(RQ1) Is it feasible to use routinely collected data within the ECC to calculate QIs that identify improvements areas orad-hocimprovements for an ED* and.
- *(RQ2) do QIs based on routinely collected data add value to the assessment of emergency care quality?*

## Methods

### Setting

Our case under consideration is a German academic teaching hospital with 771 beds treating 41,726 inpatient and 60,862 outpatient contacts in 2016. The hospital’s ED with two shock and 26 treatment rooms is open 24/7. Admissions are centrally organized to combine all patient inflows and enable a structured triage. Hence, the head of the ED has an overview of the bed occupancy and patient flow. The corresponding reports are manually conducted by station nurses. In order to support processes and manage information, the ED uses four IS listed in Table 1.

**Table 1:** Information systems used in the hospital’s ED

Type of IS	Field of application
Hospital IS	All patient charts with diagnoses and therapies
Emergency Department IS	Triage, diagnosis, and therapy within the ED
Treatment Report IS	Report on ED closures to the regional emergency control center overseeing multiple hospitals/EDs
Emergency Medical Service IS	Triage and treatment by paramedics and emergency physicians, connected to the emergency department IS

(A note that in the German healthcare system the emergency medical service is neither operated nor supervised by the hospital and that paramedics and emergency physician’s database might differ)

### Research process

Our research process comprises three steps. First, we select QIs that will be calculated within this study. Therefore, we use QIs from a systematic literature review and additionally conduct group discussions with relevant stakeholders within the hospital. Second, to answer RQ1, we combine routinely collected data extracted from various sources, preprocess it, and calculate the selected QIs. Finally, we gather our lessons learned from step two and discuss the results of the calculation and the lessons learned of our research process with subject matter experts. Based on this, we examine the added value of QIs that are based on routinely collected data to answer RQ2.

### Selection of quality indicators

As we aim to assess whether routinely collected data within the ECC can be used to calculate QIs that identify improvement potentials for an ED, we classify QIs as relevant if they are well-known in literature or demanded by practitioners and fulfill the following criteria: They 1) solely require routinely collected data for the calculation, and 2) support to identify general improvement areas or ad-hoc improvements in everyday life.

Following this, we rely on the systematic literature review of Sørup et al. from 2013 [13] for a structured overview of existing knowledge on performance and quality indicators in EDs. Their work identified a total of 55 internationally used QIs from initially 1314 articles. These articles have undergone a systematic screening resulting in 14 articles that were included for further analyses and QI extraction. Thereby, Left Without Being Seen (LWBS) and Unplanned Reattendance (UR) were identified as some of the top QIs [6, 13] to identify improvement areas, and hence, they meet our second criterion. For the first criteria, the standardized QUALIFY approach [14] suggests that UR is currently not calculable in most EDs due to missing data. However, we argue that as we aim to link routinely collected data from multiple actors within the ECC, the necessary data for UR is available. Therefore, the second criterion is met.

For the identification of QIs demanded by practitioners, we performed three group discussions with subject matter experts. Each group discussion consists of physicians working in the ED, and experts specialized in the digitization of hospitals and eHealth as well as members of the hospital’s IT department. Every participant had multiple years of work experience in her or his respective job. The group discussions were prepared in advance and followed a specific structure (i.e., the goal of the discussion, discussions on QIs, related medical processes, necessary data). Furthermore, each of the discussions had an individual focus (e.g., benefits in calculating selected QIs). All results were documented, presented to the participants for review, and approved by the participants. Based on the results, two further QIs have been selected that target the assessment of the diagnosis process and the identification of situations when EDs need to close due to crowding situations [15, 16]. Both QIs, Diagnostic Efficiency (DE) and Overload Closures (OC) [1, 17] are explicitly demanded by the practitioners of the hospital. It should be mentioned that although the indicator DE was explicitly requested by the physicians, it has not yet been conclusively validated in the literature.

Furthermore, they rely on data stored in IS that is either used by the ED, the hospital, or the treatment report. Finally, especially OC aims to support physicians in the daily decision-making process, whether the ED should be closed due to an overcrowded situation or not.

Thus, in the course of the first research step, we have selected LWBS, UR, DE, and OC as QI to be calculated in this case study. The selection is based on our previously defined criteria and the discussions with the physicians working in the ED. Hence, we do not claim that these QI are the most important or even the only relevant QIs in other settings.

After having identified our QIs (i.e., LWBS, UR, DE, and OC) during our first research step, the following step focuses on the calculation of these and starts with the preparation of the necessary data.

## Preparation

### Analysis of existing IS

Before we calculate the QIs, we first analyze the IS used within the ECC of the hospital. Therefore, we examined the four available IS regarding their actors, processes, and information technology in detail.

The primary task of emergency medical services in Germany is to avert life-threatening conditions and other severe harms to health. All emergency medical vehicles are tactically led by dispatch centers and send to the patient as required. After the prehospital stabilization, the patient is transported from the site of the accident to the hospital. In order to identify an appropriate hospital for further care provision, the dispatch center uses *IVENA*. This supra-regional web-based software shows the current care situation and availability of resources of individual hospitals.

At the arrival, a handover takes place between paramedics and ED staff. To support the handover, most emergency medical vehicles are equipped with portable devices that use *NIDA*, which is an integrated digital documentation platform to communicate with the hospital before the arrival. Thereby the paramedic sends the patients’ data to the ED, which leads to earlier coordination of all medical specialties.

**Fig. 1:**
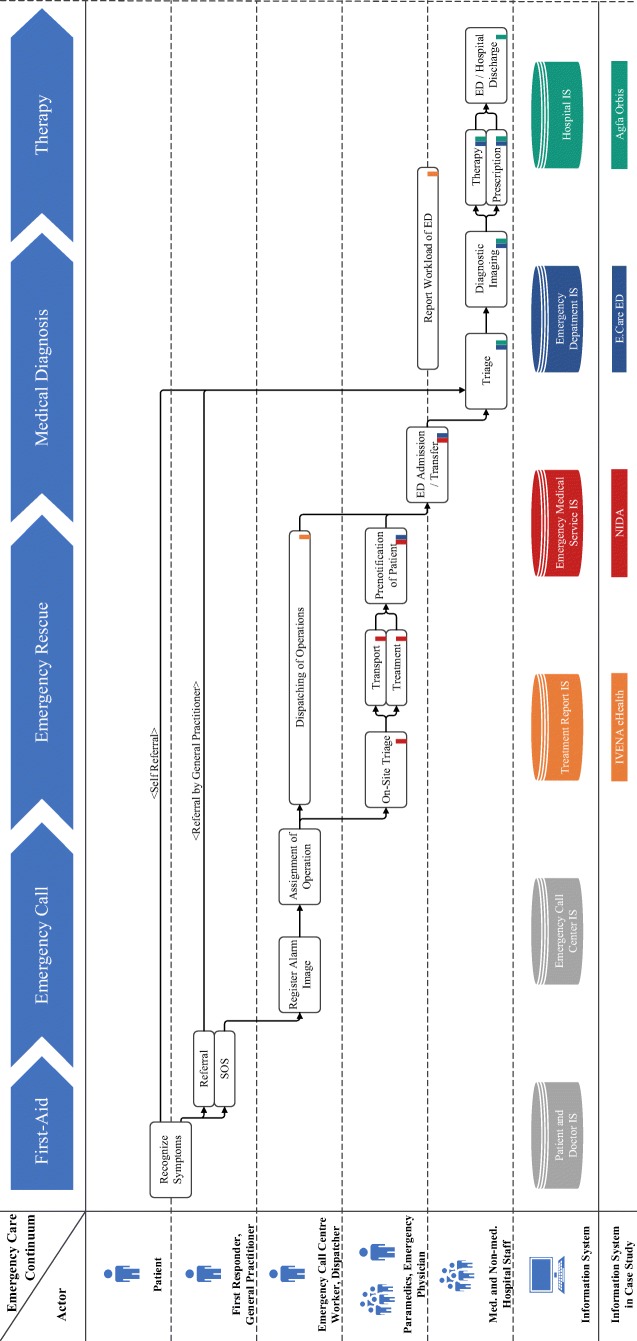
The process of data ascertainment along the chain of survival

Having arrived at the ED, the patient goes through a triage process (i.e., analyzing symptoms and vital parameters) and the first treatments are applied. To document the triage and treatment process and to manage the ED, the staff members use the emergency admission and workflow management software *E.Care*. If there is a necessity that the patient is admitted to the hospital for continuing treatment in specialist departments, the hospital’s information system *Agfa Orbis* is used. The resulting process of data detection is summarized in Fig. 1, which was iteratively developed and refined during the group discussions.

### Extraction and preprocessing of data

Having identified all available IS, we extract as well as preprocess the data to prepare the calculation. Some data is stored redundantly across multiple IS, other data (e.g., treatments in the hospital) is only available in one IS. Additionally, we observed that *NIDA* and *E.Care* already share relevant data from patient contacts. This allowed us to focus on three instead of four IS for further preparation. In the course of this, we extracted seven relations with a total of 385 data fields. We preprocessed the extracted data by removing all attributes, respectively instances (e.g., duplicates) that do not add value in order to comply with common data economy guidelines. To comply with ethical standards and existing privacy regulations, we conducted several discussions within the research team and with the hospital’s data protection officer considering potential ethical issues and especially focusing on privacy. All data handling was conducted by employees of the hospital (not the other authors) in compliance with all applicable legal boundaries and internal policies. Table 2 summarizes the extracted data relations with the corresponding amount of data fields.

**Table 2:** Breakdown of the data relations from the IS

Information system	Data relation	Description	Data fields
Hospital IS	Transfers	Transfers within the hospital (departed from ED)	29
	Discharges	Discharged patient contacts from hospital	38
	Diagnoses	Hospital admission diagnoses (equals ED discharge diagnoses) and hospital discharge diagnoses	12
Emergency Department IS	Dossiers	Patient contacts that have been admitted through paramedics (NIDA) or ED	203
	Medications	Administered medication within the ED	50
	Tasks	Treatments carried out within the ED	30
Treatment Report IS	Closures	Closures of the ED that have been reported to an emergency call center (incl. Partial closures)	23

The extracted and preprocessed data covered a total of 156,581 patient contacts in the years 2014 to 2016, while one patient can have caused multiple patient contacts within this period. In general, the patients’ demographics are comparable to other German hospitals [18]. Table 3 summarizes the relevant characteristics of all analyzed patient contacts.

**Table 3:** Breakdown of patient contact characteristics

Characteristic	Description
Age	Overall, the average age was 49.06, with a standard deviation of 26.50 years. The minimum age was 0, whereas the maximum age was 104.
Sex	50.55% of the patient contacts were labeled as “male”, 48.00% as “female”, and 1.45% of the patient contacts were unspecified.
Type of Treatment	In total, 54.31% of the analyzed patient contacts were labeled as “inpatient”, 45.50% as “outpatient” and 0.19% are not labeled.
Triage Level	0.83% of all patient contacts were labeled with triage level “red”, 21.13% with “orange”, 29.71% with “yellow”, 35.60% with “green”, 6.21% with “blue” and 6.52% were labeled as “grey” meaning there has been no triage (used e.g., for planned patient contact).

In order to link the data of the different IS, we investigate the IS’ metadata [19] and applied two different approaches. First, we identified two already existing unique identifiers that were jointly used between the Emergency Department IS and Hospital IS. While *patientID* is a unique identifier for every patient, *dossierID* is a unique identifier for every patient contact. By using *dossierID*, we were able to link all data relations from the hospital’s IS with all data relations from the emergency department’s IS. The second approach made use of the timestamps (e.g., from transfers, discharges, admissions, medications, tasks, closure start time, and closure end time) by grouping these into a predefined time frame. We were able to link two relations from the hospital IS (i.e., transfers and discharges). Each relation from the emergency department IS with the relation of the treatment report IS by dividing every day into fixed time frames (10 min in our case) and mapping all events (e.g., administered medications) that occurred within the corresponding time frame.

After preparing and linking the data, we conducted the calculation of the four QIs. In the following, we provide a short description of the QI, discuss the calculation process, and outline the results.

## Calculation

### Left without been seen

LWBS is characterized by the percentage of patients without initial care by a physician [20]. LWBS aims to avoid representations of patients with a higher urgency of symptoms who have left the ED too early or even before treatment. In order to calculate LWBS for the existing data, we analyzed a subset of 56,954 ED patients from the ED’s IS. This subset focuses on patient contacts processed by a specific group of medical specialists with the highest acceptance of providing full initial medical examination documentation. To consider patient contact where first initial care in the ED is not reasonable (e.g., forwards to other specialized hospitals), we combine this subset with transfer and discharge data from the IS of the hospital. We could identify 89.07% of the patient contacts as not left without been seen and determined LWBS with 6223 patient contacts (10.93%). However, this is only an upper boundary as the necessary data is stored within several different (free text) attributes across the ED’s and hospital’s IS. Notably, the free text attributes make a fully automatic calculation hardly possible.

### Unplanned reattendance

UR represents the percentage of unscheduled patients returning to the ED with comparable symptoms within 72 h [21, 22]. This indicator aims to identify overlooked illnesses or injuries of previously treated patients. It must, therefore, be distinguished whether a reattendance is caused by mistake by the ED or by other care providers. Additionally, scheduled reattendances (e.g., for post-treatments, hence not unplanned) or reattendances outside the 72 h time frame were excluded. Hence, we analyze transfers and discharges by combining information from the IS of the emergency department and the hospital. A total of 23,038 reattending ED patient contacts were identified, of which 2281 were only treated by the ED. Additionally, anonymized demographic patient information, stored in the hospital IS, was attached to have a better understanding of UR patients and to enable identification of potential risk groups. 2281 out of 156,581 ED patient contacts (1.46%) have been identified as ED-based UR. Compared to our study population, the average age of patients classified as unplanned reattendance is 8.04 years lower. 96.36% of the reattendances were classified as not life-threatening or organ-damaging [23, 24].

### Diagnostic efficiency

DE focuses on the quality of the diagnosis process [1, 25] and aims to identify efficient and inefficient treatment cases. This information is used to apply purposeful measures (e.g., staff training, purchase of additional or better equipment) in order to improve the recognition rate of critical diseases (e.g., acute myocardial infarction (I21), intracranial injury (S06)) by [25]. Data to calculate all relevant information (length of stay and diagnostic agreement) is stored across different data sets [26] with a huge difference regarding data quality. For example, the ICD-10-GM-encoded diagnoses are carefully entered into the hospital’s IS by the staff of the ED. In contrast, other actors carelessly use the IS of the ED, which results in unrealistic length-of-stay values. For our study, we calculate the DE for 28 diagnoses that require immediate treatment and therefore are highly relevant for EDs [1, 25]. In an initial step, we calculate diagnostic agreement as to the accordance of the main diagnosis at the patient’s hospital admission (equals in our case the discharge of the ED) with the main diagnosis documented on the patient’s discharge of the hospital. Both diagnoses are ICD-10-GM-encoded in three alphanumeric digits and stored in the hospital IS. The length of stay used to calculate the DE is derived by calculating the average length of stay per diagnosis for each patient contact stored in the emergency department IS with the corresponding diagnosis. Table 4 lists five exemplary diagnoses that were calculated. In general, the calculation of DE is possible and results in reasonable scores that can be further evaluated by medical experts. Some scores, e.g., for I71, seem to be wrong since outliers in the length of stay caused by bad data quality may have profoundly influenced the results.

**Table 4:** Calculated diagnostic efficiency for five ICD-10 diagnoses based on routinely collected data

ICD-10-GM code	Diagnostic agreement	Length of stay (minutes, avg.)	Diagnostic efficiency
G45 + I63 (*n* = 2900)	0.442	96.149	0.459
I21 (*n* = 1406)	0.536	102.127	0.524
I71 (*n* = 57)	0.561	1018.193	0.006
J12 – J18 (*n* = 2670)	0.467	103.051	0.453
S06 (*n* = 1896)	0.943	106.117	0.889

### Overload closure

OC aims to predict upcoming situations when EDs might need to close due to overload. In Germany, heads of EDs legally have to continuously evaluate whether the current number of patients waiting for treatment can be handled with the available resources in a reasonable time, providing a sufficient level of quality. A QI that indicates whether a potential overload situation comes up helps them to take appropriate measures at an early stage (e.g., to stop the admission of new patients, requesting additional personnel resources). First, we analyzed the available data within the ECC of the hospital in order to exploratively identify relevant attributes helping to estimate the workload within the ED. Based on intensive discussion with physicians, we selected the following attributes as potential signals for an overload situation: number of administered medications, number of performed tasks, waiting time, and triage level of the patient contact. We used different time frames to aggregate these criteria. Also, we applied conventional data analytics methods to classify whether the ED has stopped the admission of new patients, which is stored in the treatment report IS. In order to build a prediction model, three common types of classifiers were used: logistic regression, multilayer perceptron, and decision tree [27–29]. We found that the best classifier, a C4.5 algorithm-based decision tree, has an accuracy of 90.17% (recall “opened ED”: 98.2%; recall “closed ED”: 82.1%). Our final model considers the waiting time and the triage level of the patients in an eight-hour timeframe as the relevant indicators for a potential overload situation.

## Conclusion

### Discussion

All four QIs enables the hospital to identify areas of improvement regarding the EDs structure or routines, and especially OC supports physicians in everyday life. For example, we run into the issue that we were not able to determine the exact value of LWBS but were able to identify a possible explanation for this and then take appropriate measures. A staff member of the ED reported that some medical specialists are used to enter patient information into the hospital IS and are not willing to document the full initial medical examination in the emergency department IS. This shows us that QIs that could generally be easily calculated cannot be calculated due to a lack of availability of the necessary data or due to insufficient data quality.

In order to answer the question of whether QI based on routinely collected data add value, we will further present our lessons learned and extend this with the results of additional expert interviews.

In general, we can divide our lessons learned into two different topics. Our first lesson learned topic is that the use of routinely collected data increases the efficiency and effectiveness of the calculation process. On the one hand, this means that the indicators can be calculated faster or with less effort, as no manual steps or additional data collections are necessary, and preprocessing steps can be automated. However, this is only possible if the quality of the routinely collected data is sufficient. On the other hand, it is possible to add further contextual information enabling a better understanding of the QIs and, thus, support the identification of improvement areas. For example, by calculating UR and using other additional contextual information (e.g., age, gender, triage-level), we gain insights into unplanned-reattendance-prone patient demographics. We can better understand their medical condition urgency, which allows decision-makers in ED to tailor targets to the specific context and supports the implementation of measures. However, the calculation of QIs based on routinely collected data can also entail risks and requires the results to be critically examined. Although DE enables a better understanding of the trade-off between time constraints and effective diagnosis, the QI is quite sensitive to data quality issues, due to the critical role of the length of stay in the formula. This could, in turn, leads to a wrong interpretation of the indicator and hence, the implementation of wrong measures. A possible solution in the case of DE could be to apply further data pre-processing steps (i.e., removing outliers). It should be noted that the classification as outlier heavily depends on the examined diagnosis and should, therefore, not be performed without the consideration of medical expertise. This underlines the need for a continued development and validation of this indicator.

Our second lesson learned topic is that using routinely collected data also makes it possible to calculate new QI that supports physicians in their decision-making-process in everyday life. An example of this was the prediction of whether an ED should be opened or closed for new patients. Although it still requires experienced ED staff members to interpret the QI and make the decision, the medical quality officer is surprised about the accuracy of the developed prediction model. He argues that this QI enables them to timely take appropriate measures in order to prevent potential overload situations.

In general, all experts noted that the calculation of QIs using data pools within the ECC offers excellent potential for quality improvement collaboratives for prompt and informed decision making. Furthermore, they outline that this may only be the first step and that the case study only touches the surface of possible opportunities to increase the quality of an ED using routinely collected data. Despite significant quality improvement efforts to accelerate the fostering of emergency care, there are still treatment delays and failures, which could be eliminated through gaining a comprehensive understanding of routinely collected data within the quality improvement collaborative.

### Limitations

The results of this study should be interpreted with consideration of the following limitations. First, we faced some issues during the calculation of the QIs. We only calculated four QIs that were selected by our literature search and the experts of the hospital. Even though we aimed to have a mixture of various types of QI, there exist many further QIs in literature. Second, since we calculated our QI in one teaching hospital in Germany evaluated by experts of this hospital, the external validity of our results is rather low. Although the academic teaching hospital is comparable to other hospitals in Germany in terms of its study population, it is also quite innovative with an almost paperless emergency department.

### Summary and future work

We calculated different QIs within a single case study in a German academic teaching hospital in order to answer our research questions. Finally, lessons learned from our research provide a better understanding of the quality of the ED, the related challenges during the calculation, and the added value of linking routinely collected data. Besides this, our research contributes by identifying different types of IS and data pools used in Germany within the ECC. Furthermore, to the best of our knowledge, it was the first time that DE was calculated outside of a clinical study. One reason why DE has so far only been calculated in clinical studies could be that the indicator is difficult to calculate in practice, as the necessary information is spread across several IS. A linking of data, as in our case study, will also allow further research on DE to be facilitated and, thus, help to mature and internationally validate the indicator.

Our research shows the potential of QIs based on routinely collected data from multiple IS within the ECC and paves the way for improved quality in EDs. Hence, the consistent networking of actors within the ECC has the potential to develop new measuring instruments and to depict the reality of care in a more targeted manner in order to improve the quality of care ultimately.
